# Deep-Learning-Based Group Pointwise Spatial Mapping of Structure to Function in Glaucoma

**DOI:** 10.1016/j.xops.2024.100523

**Published:** 2024-04-02

**Authors:** Zhiqi Chen, Hiroshi Ishikawa, Yao Wang, Gadi Wollstein, Joel S. Schuman

**Affiliations:** 1 Department of Electrical and Computer Engineering, NYU Tandon School of Engineering, Brooklyn, New York; 2 Department of Ophthalmology, NYU Langone Health, NYU Grossman School of Medicine, New York, New York; 3 Department of Ophthalmology, Casey Eye Institute, Oregon Health and Science University, Portland, Oregon; 4 Department of Medical Informatics and Clinical Epidemiology, Oregon Health and Science University, Portland, Oregon; 5 Department of Biomedical Engineering, NYU Tandon School of Engineering, Brooklyn, New York; 6 Center for Neural Science, NYU College of Arts and Sciences, New York, New York; 7 Glaucoma Service, Eye Hospital, Philadelphia, Pennsylvania; 8 Department of Ophthalmology, Sidney Kimmel Medical College at Thomas Jefferson University, Philadelphia, Pennsylvania; 9 Drexel University School of Biomedical Engineering, Sciences and Health Studies

**Keywords:** Structure-to-function mapping, Deep learning, Structure-function relationship, VF, Glaucoma

## Abstract

**Purpose:**

To establish generalizable pointwise spatial relationship between structure and function through occlusion analysis of a deep-learning (DL) model for predicting the visual field (VF) sensitivities from 3-dimensional (3D) OCT scan.

**Design:**

Retrospective cross-sectional study.

**Participants:**

A total of 2151 eyes from 1129 patients.

**Methods:**

A DL model was trained to predict 52 VF sensitivities of 24-2 standard automated perimetry from 3D spectral-domain OCT images of the optic nerve head (ONH) with 12 915 OCT-VF pairs. Using occlusion analysis, the contribution of each individual cube covering a 240 × 240 × 31.25 μm region of the ONH to the model's prediction was systematically evaluated for each OCT-VF pair in a separate test set that consisted of 996 OCT-VF pairs. After simple translation (shifting in x- and y-axes to match the ONH center), group t-statistic maps were derived to visualize statistically significant ONH regions for each VF test point within a group. This analysis allowed for understanding the importance of each super voxel (240 × 240 × 31.25 μm covering the entire 4.32 × 4.32 × 1.125 mm ONH cube) in predicting VF test points for specific patient groups.

**Main Outcome Measures:**

The region at the ONH corresponding to each VF test point and the effect of the former on the latter.

**Results:**

The test set was divided to 2 groups, the healthy-to-early-glaucoma group (792 OCT-VF pairs, VF mean deviation [MD]: −1.32 ± 1.90 decibels [dB]) and the moderate-to-advanced-glaucoma group (204 OCT-VF pairs, VF MD: −17.93 ± 7.68 dB). Two-dimensional group t-statistic maps (x, y projection) were generated for both groups, assigning related ONH regions to visual field test points. The identified influential structural locations for VF sensitivity prediction at each test point aligned well with existing knowledge and understanding of structure-function spatial relationships.

**Conclusions:**

This study successfully visualized the global trend of point-by-point spatial relationships between OCT-based structure and VF-based function without the need for prior knowledge or segmentation of OCTs. The revealed spatial correlations were consistent with previously published mappings. This presents possibilities of learning from trained machine learning models without applying any prior knowledge, potentially robust, and free from bias.

**Financial Disclosure(s):**

Proprietary or commercial disclosure may be found in the Footnotes and Disclosures at the end of this article.

Glaucoma is a progressive optic neuropathy characterized by a distinct pattern of structural and functional damage. Structural damage encompasses harm to the retinal ganglion cells, their axons, and associated glial cells, resulting in noticeable changes in the appearance of optic nerve head (ONH). These changes are commonly assessed using advanced imaging techniques such as OCT. Functional damage involves a loss of light sensitivity, often measured through visual field (VF) loss using standard automated perimetry. Both structural and functional damage provide crucial information for glaucoma diagnosis as well as its management. Although there is a strong correlation between structural and functional measurements, there are many clinical cases that cannot be clearly explained with such a simple correlation. Since glaucoma progression patterns widely vary from individual to individual, a detailed spatial correlation map may help identifying personalized progression pattern and provide better assessment and forecasting of progression.

Structure-function spatial correlation has been widely investigated.^1^, ^2^, ^3^, ^4^, ^5^ Perhaps the most well-known depiction is the Garway-Heath map that associates clusters of VF test points with sectors of the optic disc by superimposing 24-2 VF test grid on retinal photographs and manually tracing visible retinal nerve fiber layer (RNFL) defects or prominent nerve fiber bundles to note their point of intersection.^1^ The derived map divides the ONH and 24-2 VF into 6 corresponding sectors. Jansonius et al^2^ later proposed a mathematical model fitting hand-traced retinal nerve fiber trajectories to reduce variabilities in hand-tracing, leading to a more robust portrayal of the structure-function relationship. Alternative approaches used statistical methods to produce the structure-function correspondence. Gardiner et al^3^ utilized maximum correlation between normalized rim area of 36 sectors measured by Heidelberg retina tomography and 24-2 VF sensitivities. Turpin et al^4^ further constrained the correlation between Heidelberg retina tomography measurements and VF to be anatomically plausible with a computational model of the axon growth of retinal ganglion cells. Ferreras et al^5^ used factor analysis to divide 24-2 VF grid into 10 sectors. Then, a similar correlation approach was applied to relate predefined 10 VF sectors to clock-hour sectors of peripapillary RNFL thickness measured by OCT.

All previous studies either are based on prior knowledge regarding anatomic structures and their functions or require segmentations to get ONH measurements. It can certainly be a good way of establishing the structure–function relationships. However, it is possible to discover unexpected anatomic or structural features that are highly associated with function using artificial intelligence. Recent advances in deep-learning (DL) approaches achieve unprecedented performance—sometimes better than human experts—in many medical applications. While DL models are known to be black boxes, recently many techniques to reveal which location within the input image contributed the most to reach the output have been developed. In other words, it is now possible to learn from well-trained DL models.

Several previous studies have attempted to predict VF outcomes using OCT measurements through DL algorithms.^6^, ^7^, ^8^, ^9^, ^10^, ^11^, ^12^, ^13^, ^14^, ^15^, ^16^, ^17^ Although these studies have shown promising results in approximating VF metrics from OCT data, the precise spatial relationship between structural damage and functional damage remains less well-established. Mariottoni et al^7^ created a mapping between the 768-point RNFL thickness profile obtained from a spectral-domain OCT peripapillary scan and the 24-2 standard automated perimetry VF loss by simulating localized RNFL defects of varying locations and characteristics. They observed the impact of these defects on VF outcomes using a convolutional neural network designed to predict VF sensitivities from RNFL thickness profiles. The derived map offers a more detailed spatial structure–function relationship compared to the Garway-Heath map, but their method depends on the segmentation outcomes, which can be affected by image quality and segmentation errors.^18^ Kihara et al^16^ proposed a multimodal policy DL system that directly predicts VF from unsegmented circumpapillary OCT and scanning laser ophthalmoscopy (SLO) image of the ONH. Thus, a circumpapillary sector structure–function mapping was derived in a data-driven, feature agnostic fashion. Nonetheless, all prior mappings remained limited to sector representations, which is suboptimal as they fail to fully exploit the 3-dimensional (3D) nature of retinal structure. A more comprehensive spatial mapping, derived from 3D structure measurements (e.g., 3D OCT data) and independent of domain-specific knowledge (e.g., segmented RNFL thickness), is desired to enhance our understanding of the spatial relationship between structure and function.

Recently, DL algorithms have ventured into analyzing higher-dimensional data to leverage 3D information that may not be readily discernible through conventional methods.^6^ Consequently, in this study, we aim to establish a generalized pointwise spatial mapping between structure and function by conducting occlusion analysis on a DL model trained on an extensive clinical cohort of patients to predict pointwise VF sensitivities from 3D OCTs. To the best of our knowledge, this is the first work generating a group saliency map using 3D OCT data without segmentation around the ONH aiming for establishing point-by-point (VF) structure–function mapping.

## Methods

### Data Collection

This was a retrospective cross-sectional study. The institutional review board of the New York University Langone Medical Center approved this study, and a waiver of informed consent was granted owing to the retrospective nature of this work. All methods adhered to the tenets of the Declaration of Helsinki for research involving human participants, and the study was conducted in accordance with regulations of the Health Insurance Portability and Accountability Act.

Subjects were included in the study according to the following inclusion criteria: ≥1 reliable VF test and 1 reliable 3D spectral-domain OCT data within 90 days of each other. Visual field tests were performed using the Humphrey Field Analyzer with the 24-2 Swedish Interactive Threshold Algorithm (Zeiss) standard protocol. A reliable VF test has fixation losses, false-positive errors, and false-negative errors <33%, 15%, and 15%, respectively. Spectral-domain OCTs were acquired by the Cirrus HD-OCT instrument (Zeiss) using the 6 × 6 mm ONH scan 200 × 200 protocol. A reliable test has signal strength >6 decibels (dB).

**Figure 1:**
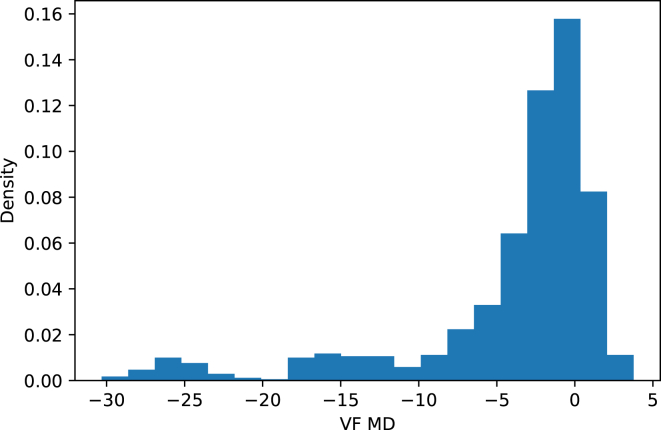
Distribution of VF MD. MD = mean deviation; VF = visual field.

The final data set comprised 8015 VF tests and 15 026 ONH OCT scans from 1108 subjects spanning multiple visits. The distribution of VF mean deviation (MD) is shown in Figure 1. We randomly split the data set at a ratio of 9:1 based on subjects to create training and test sets. Consequently, the training set contained 7303 VF tests and 10 711 ONH scans from 999 subjects. Every OCT was associated with every possible VF test that was within 90 days of the OCT visit, resulting in 12 915 OCT-VF pairs in total for training. The test set contained 996 OCT-VF pairs from 247 eyes (145 female + 102 male, 180 White + 63 Black + 2 Asian + 2 unknown). All participants were clinically diagnosed with glaucoma, glaucoma suspect, or healthy after undergoing a comprehensive ophthalmic evaluation that included a clinical examination, a VF testing, and an OCT. Among them, 121 eyes have glaucoma (108 open-angle + 11 closed-angle + 2 mixed-mechanism), 108 eyes are glaucoma suspect, and 18 eyes are healthy. Every OCT and VF visit in the test set was unique, that is, 1 OCT was only associated with 1 VF test. Table 1 summarized the demographic characteristics of the data set.

**Table 1:** Demographics of the Data Set

	Training Set	Test Set
Number of subjects	999	130
Number of eyes	1904	247
Number of OCT-VF pairs	12 915	996
Age at testing date (year)	64.45 ± 12.69 (18 ∼ 94)	62.39 ± 11.59 (18 ∼ 86)
VF MD (dB)	−4.69 ± 6.86 (−32.78 ∼ 5.78)	−4.05 ± 6.47 (−30.32 ∼ 3.77)

dB = decibels; MD = mean deviation; VF = visual field.

To preprocess the OCTs and VFs, we adopted the same preprocessing steps as described in the study by Chen et al.^6^ In brief, for OCT, we detected the ONH region and flattened Bruch’s membrane opening surface by segmenting Bruch’s membrane opening with smoothing and moving each A-scan along the z direction. Then central cropping and downsampling with gaussian antialiasing filtering were applied to reduce memory consumption during model training. After preprocessing, all 3D ONH OCTs were flattened by Bruch’s membrane opening, centrally cropped at the optic disc center to 144 × 144 x 576 voxels (covers a 4.32 × 4.32 × 1.125 mm region of ONH), and downsampled to 72 × 72 × 144 voxels. For VF, the 2 blind spot points were excluded. The sensitivities were temporally smoothed over 5 consecutive VF visits (average time span was 1166.04 ± 598.91 days) of the same eye using pointwise linear regression to reduce random fluctuations. In average, each eye has 9.16 ± 3.90 VF visits used in temporal smoothing. For eyes that had <5 VF visits, we used the original VFs. All left eye visits were flipped horizontally to match the right eye format for both OCTs and VFs.

### Model Architecture and Training

Since the purpose of this study was to derive the spatial relationship between structure and function, rather than developing a new model to predict function from structure, we adopted the same model, a 3D convolutional neural network, from the study by Chen et al,^6^ which showed promising performance in predicting standard automated perimetry sensitives from 3D OCT data of the ONH.

**Figure 2:**
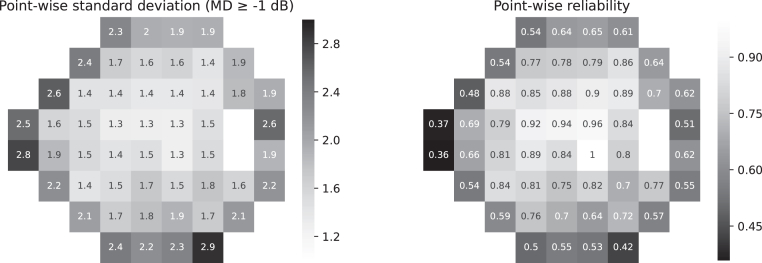
Pointwise reliabilities. dB = decibels; MD = mean deviation.

We adopted the same training strategy as used in the study by Chen et al,^6^ that is, we trained the model with the Adam optimizer^19^ for 200 epochs and a batch size of 16. The learning rate was initially set to 2 × 10^−4^ and linear decayed every 100 epochs by 10^−1^. Different from the study by Chen et al,^6^ we used a reliability-weighted mean square error loss function instead of standard mean square error loss to compensate for larger noises in peripheral VF test points. The pointwise weight of the loss function, indicating the reliability of VF measurements, was defined as the inverse of the pointwise standard deviation of VF sensitivities among healthy subjects (VF MD >−1 dB), with the highest reliability normalized to 1. The weight for each VF test point is shown in Figure 2. The proposed reliability-weighted loss function improved the average mean absolute error from 3.11 dB as reported in ^6^ to 2.99 dB.

### Structure–Function Mapping

Once an accurate model is established to estimate pointwise VF sensitivities from 3D OCT images, it becomes possible to derive spatial correlations between each test point of the VF and the corresponding regions of the ONH using the model. To establish this spatial mapping, we first applied occlusion analysis on the trained model, generating 52 pointwise 3D saliency data/volume (in this article, we use volume and 3D data interchangeably) for every sample in the test set. This allowed us to evaluate the contribution of individual regions in the input OCT volume to the model's predictions. To ensure consistency, all saliency volumes were registered using the geometric center of the optic disc. Additionally, to account for variations along the depth dimension, we averaged each saliency volume across depth to generate a 2-dimensional (2D) individual saliency map, analogous to OCT en face images.

Next, in order to generate group saliency maps for specific glaucoma groups, we divided the test set into the healthy-to-early-glaucoma group and the moderate-to-advanced-glaucoma group based on VF MD values (cutoff at MD −6 dB). A pointwise *t* test was performed separately for each small ONH region within each group. This enabled us to generate group t-statistic maps (aka group saliency map), revealing the detailed spatial relationship between each VF test point and the corresponding statistically significant and relevant regions of the ONH for a specific glaucoma group.

#### Individual Saliency Map by Occlusion Analysis

Occlusion analysis is widely used to visualize the decision-making process of black box models. In this study, we utilized occlusion analysis to quantify the contribution, also known as saliency, of each small region of the ONH on model’s prediction for each VF test point. The underlying assumption is that if a region of the ONH is related to a VF test point, removing information from that region will significantly alter the DL model's prediction for the corresponding point. Conversely, the model's prediction should remain consistent when removing information from irrelevant ONH regions.

To implement this, we replaced a small region (4 × 4 × 4 voxels, 240 × 240 × 31.25 μm) within an input volume with a gray patch (mean intensity of the input) and calculated the saliency by comparing the model's prediction with the original input to its prediction with the occluded input. The saliency was defined as the absolute difference between the model predictions for the original and occluded inputs. By repeating this process for all locations throughout the entire input volume for all VF test points of each ONH volume in the data set, we generated 52 saliency volumes for every OCT-VF pair in the data set. Specifically, for each OCT volume $V_{i}$ in the test set $\left\{V_{1},V_{2},\ldots ,V_{N}\right\}$, we obtained 52 saliency volumes $\left\{S_{i,1},S_{i,2},\ldots ,S_{i,52}\right\}$ corresponding to the 52 VF test points. Each voxel $S_{i,pt}^{j}$ within a saliency volume $S_{i,pt}$ represented the absolute difference between the model predictions when occluding the j-th small region of the ONH volume:

$$S_{i,pt}^{j}=|f\left(V_{i}\right)-f\left(\;T^{j}\left(V_{i}\right)\;\right)|$$

where $V_{i}$ denotes the original ONH volume, and $T^{j}\left(\cdot \right)$ denotes the operation that replaces the *j-th* patch of $V_{i}$ with a value equal to the volume's mean intensity ${\bar{V}}_{i}$. An example of a 3D saliency volume was presented in the Appendix (Figs 7 and 8).

**Figure 3:**
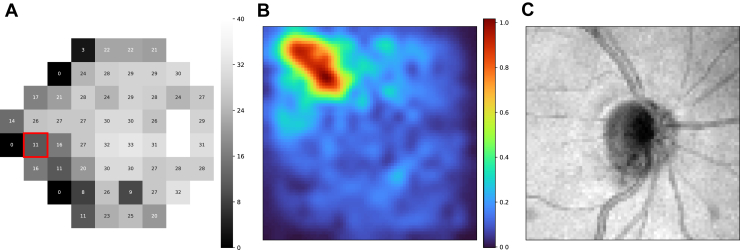
An example of an individual saliency map of a particular VF test point. **A,** VF sensitivities of a subject in the test set. **B,** The saliency map of a particular test point (highlighted with a red bounding box in [**A]**). **C,** the corresponding en face OCT image. VF = visual field.

**Figure 4:**
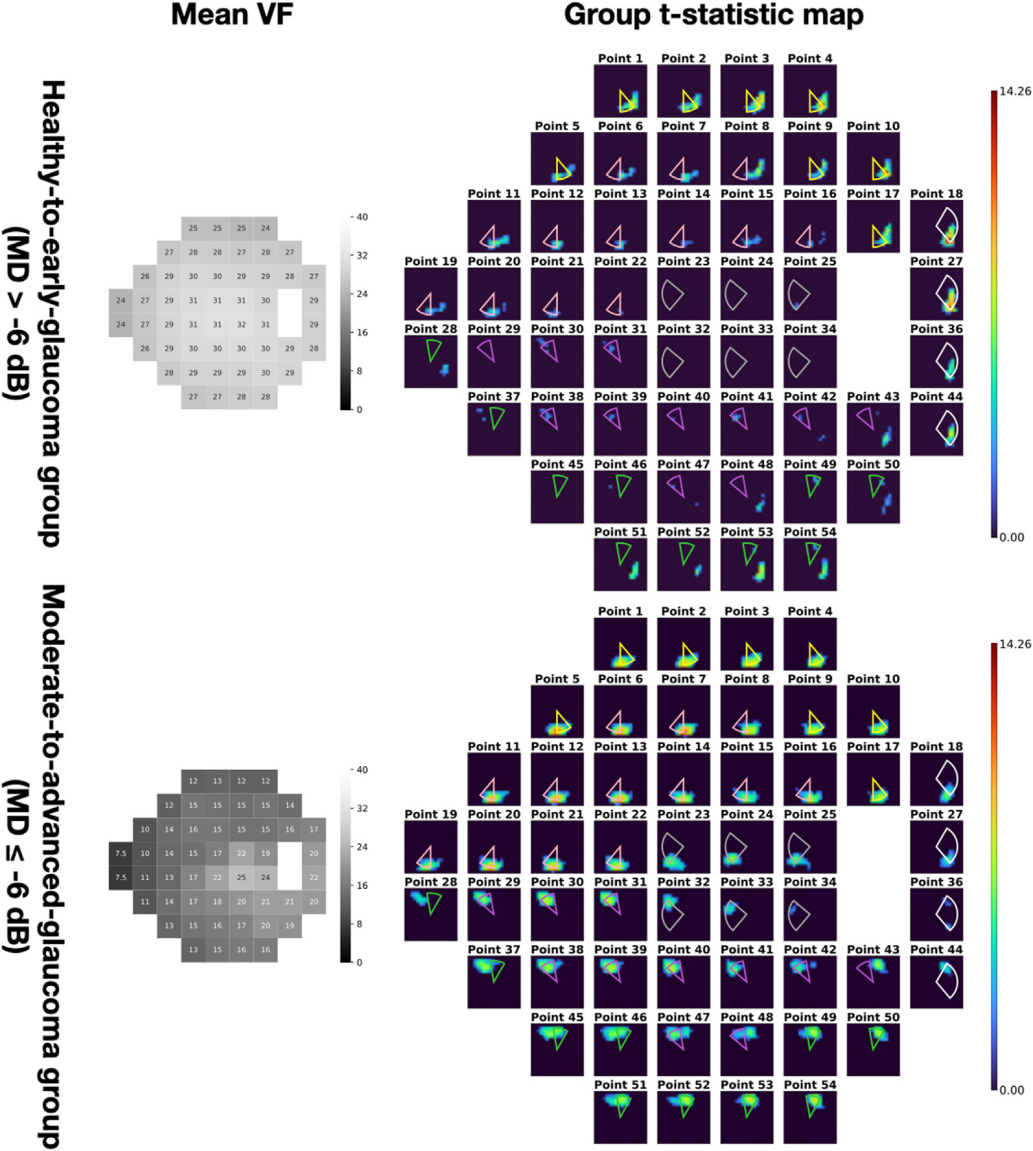
Group t-statistic maps for the healthy-to-early-glaucoma group (MD >−6 dB) and the moderate-to-advanced glaucoma group (MD ≤−6 dB). ONH sectors proposed by the Garway-Heath map were overlaid on top for comparison. Different colors represent different VF clusters defined in the Garway-Heath map. dB = decibels; MD = mean deviation; ONH = optic nerve head; VF = visual field.

**Figure 5:**
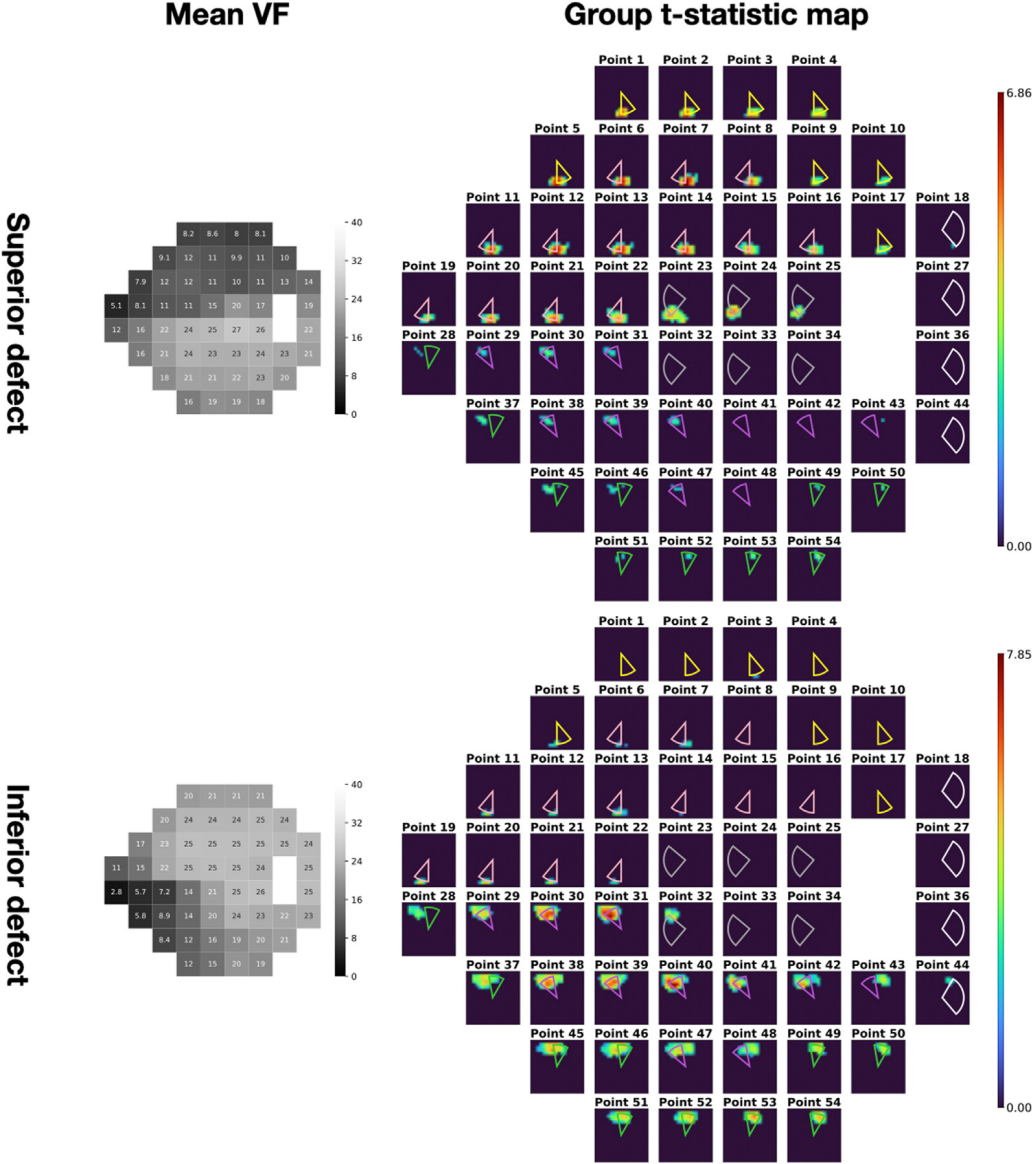
Group t-statistic maps of superior and inferior defects. ONH sectors proposed by the Garway-Heath map were overlaid on top for comparison. Different colors represent different VF clusters defined in the Garway-Heath map. ONH = optic nerve head; VF = visual field.

**Figure 6:**
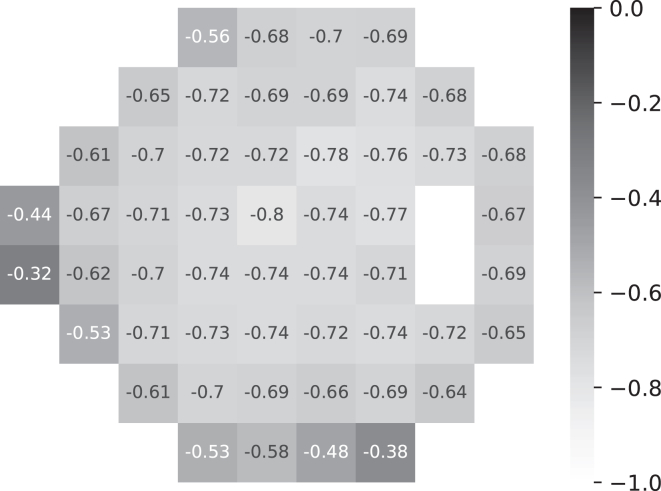
Pointwise Pearson's correlation between saliency magnitude and VF MD. MD = mean deviation; VF = visual field.

**Figure 7:**
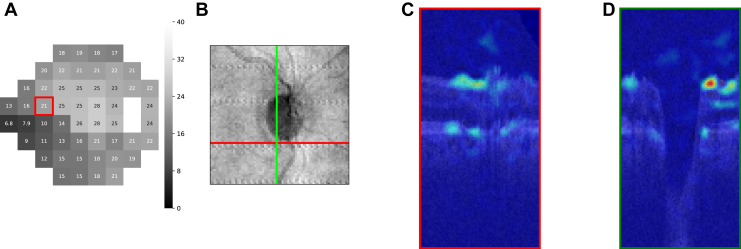
The cross-sectional view of an individual saliency volume. **A,** A VF test. **B,** The associated en face OCT image. **C,** Cross-sectional B-scan associated with the red line in (**B**), overlaid with the corresponding saliency of point 21 highlighted with red bounding box in (**A**). **D,** Cross-sectional B-scan associated with the green line in (**B**), overlaid with the corresponding saliency of point 21 highlighted with red bounding box in (**A**). VF = visual field.

**Figure 8:**
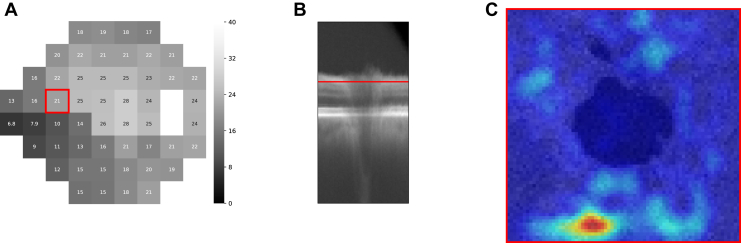
The *en-face* view of an individual saliency volume. **A,** A VF test. **B,** The associated cross-sectional OCT B-scan. **C,** A scan associated with the red line in (**B**), overlaid with the corresponding saliency of point 21 highlighted with red bounding box in (**A**). VF = visual field.

To account for variation across depth due to the adopted coarse registration, the 3D individual saliency volumes $\left\{\left\{S_{1,1},S_{1,2},\ldots ,S_{1,52}\right\},\left\{S_{2,1},S_{2,2},\ldots ,S_{2,52}\right\},\ldots ,\left\{S_{N,1},S_{N,2},\ldots ,S_{N,52}\right\}\right\}$ were projected onto the en face plane, generating 2D individual saliency maps $\left\{\left\{\;S_{1,1}^{\prime },S_{1,2}^{\prime },\ldots ,S_{1,52}^{\prime }\right\},\left\{S_{2,1}^{\prime },S_{2,2}^{\prime },\ldots ,S_{2,52}^{\prime }\right\},\ldots ,\left\{S_{N,1}^{\prime },S_{N,2}^{\prime },\ldots ,S_{N,52}^{\prime }\right\}\right\}$ to address depth-related variations and to ease visualization.

#### Group Saliency Map by *t* Test

To identify statistically significant and relevant ONH regions for each VF test point in a specific glaucoma group, we divided the test set into 2 groups, the healthy-to-early-glaucoma and the moderate-to-advanced-glaucoma groups, and separately conducted *t* tests within each group. This process yielded the corresponding group t-statistic maps, which encode the group-specific spatial relationships between structure and function.

The group t-statistic maps were generated by separately performing *t* tests for each small ONH region, comparing its saliency with the pointwise group-averaged saliency. For a particular 4 × 4 region *k* in the en face plane and a particular VF test point *pt*, we had $\left\{{S^{\prime }}_{1,pt}^{k},{S^{\prime }}_{2,pt}^{k},\ldots ,{S^{\prime }}_{N,pt}^{k}\right\}$ representing the saliency of region *k* for model prediction at VF test point *pt* for each subject in a group. The group t-statistic map $T_{pt}$ of a particular VF test point *pt* was created by conducting *t* test separately across all regions of ONH:

$$T_{pt}^{k}=\left\{ \begin{array}{c} \frac{{\bar{S}}_{\;pt}^{k}-\;\mu_{pt}}{\sigma_{pt}^{k}/\sqrt{N}},if\;\alpha \leq 0.05\\ 0,if\;\alpha >0.05 \end{array}\right.$$

where ${\bar{S}}_{\;pt}^{k}$ and $\sigma_{pt}^{k}$ denote the sample mean and sample standard deviation of saliencies for a particular patch *k*, and $\mu_{pt}$ denotes the hypothesis mean. We set $\mu_{pt}$ to be ${\bar{S}}_{\;pt}+\lambda \sigma_{pt}$, where λ=0.75. In other words, 1-sample *t* test was conducted to determine the extent to which the mean saliency of a particular patch *k* for a particular test point *pt* exceeds the mean saliency averaged over all patches ${\bar{S}}_{\;pt}$ (adjusted by standard deviation $\sigma_{pt}$ as well) within the same group for that test point *pt*.

As a result, this study generated a new map, namely the group t-statistic map, which establishes the spatial relationship between each VF test point and the corresponding significantly relevant regions of the ONH.

## Results

All results shown in this study were generated on the test set. There were 792 OCT-VF pairs from 207 eyes for the healthy-to-early-glaucoma group (MD >−6 dB, mean MD −1.32 ± 1.90 dB) and 204 pairs from 66 eyes for the moderate-to-advanced glaucoma group (MD ≤−6 dB, mean MD −17.93 ± 7.68 dB). Figure 3 shows an example of an individual saliency map that corresponds to a particular VF test point. Figure 4 shows the mean VF sensitivity maps and the groups t-statistic maps for both groups (also, Appendix Fig 9). Optic nerve head sectors proposed by the Garway-Heath map were overlaid on top of the group t-statistic maps for comparison. In general, our maps showed good agreement with the Garway-Heath map. Note that we have depth information that is not depicted in the en face expression of the maps (please see Figs 7 and 8 in the Appendix for an illustration). Therefore, we have not only x- and y-axes but also have z-axis information, which might potentially improve the mapping.

**Figure 9:**
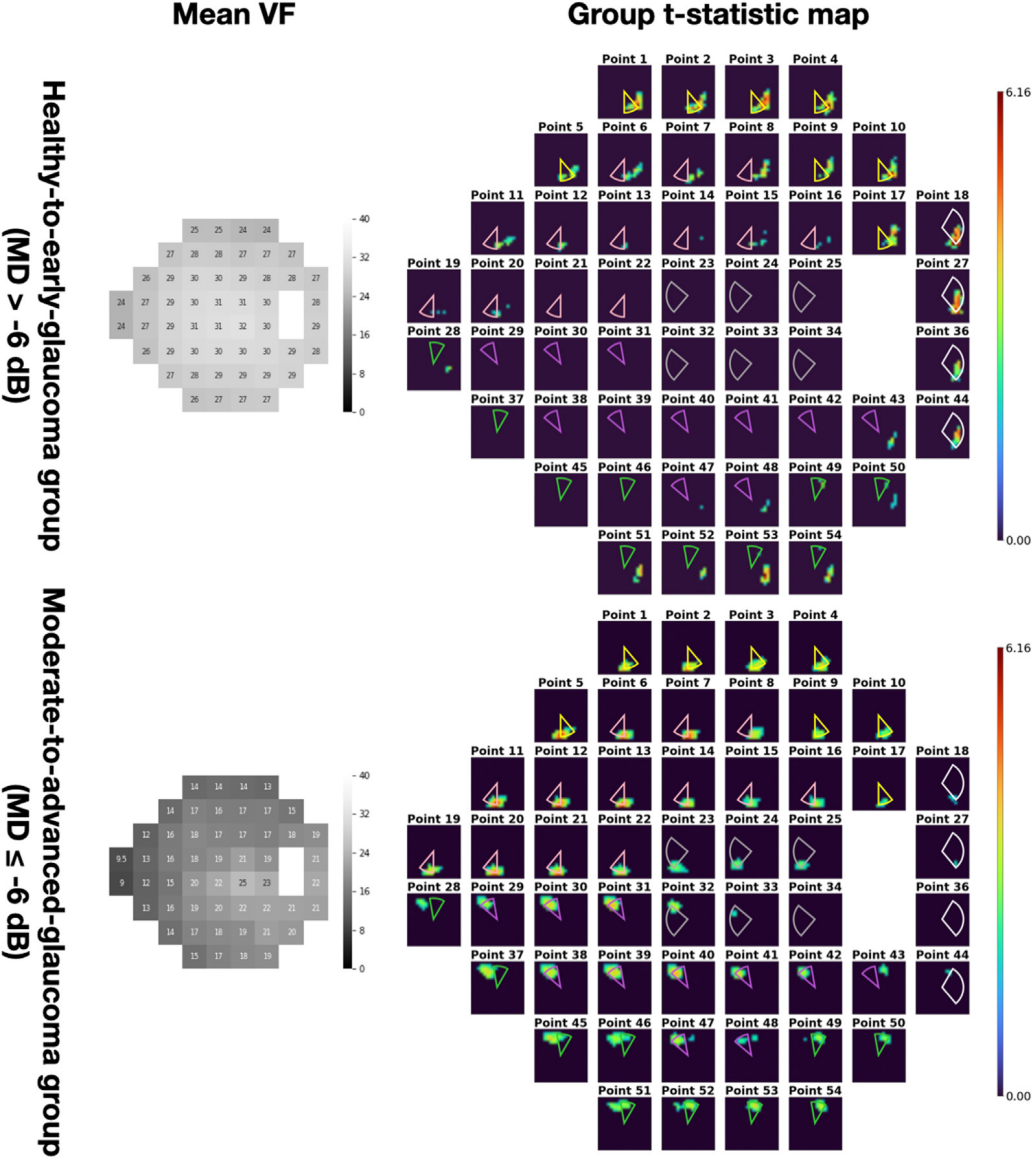
Group t-statistic maps for the healthy-to-early-glaucoma group and the moderate-to-advanced-glaucoma group (resampled to avoid within-subject correlation). ONH sectors proposed by the Garway-Heath map were overlaid on top for comparison. Different colors represent different VF clusters defined in the Garway-Heath map. dB = decibels; MD = mean deviation; ONH = optic nerve head; VF = visual field.

We further selected 2 subsets, the superior and inferior defect groups, from the moderate-to-advanced glaucoma group by manually observing the 24-2 VF defect patterns. The superior defect group included 74 OCT-VF pairs and the inferior group included 40 OCT-VF pairs. The corresponding VF sensitivity maps and group t-statistic maps are shown in Figure 5. Similarly, Garway-Heath sectors were overlaid on the t-statistic maps for comparison. The 2 groups’ t-statistic maps showed symmetric patterns for superior and inferior damage groups. That is, the groups’ t-statistic map of superior damages highlighted the inferior part of retina and vice versa for the map of inferior damages.

Figure 6 depicted pointwise negative Pearson's correlation between saliency and VF MD, indicating a stronger relationship with more severe defects.

## Discussion

In this study, we successfully generated a generalized 2D mapping that establishes the group spatial relationship between VF test points and regions of the ONH at a fine resolution. Importantly, our algorithm relied solely on the data without any prior knowledge about the structure–function relationship, free from potential bias, segmentation errors, and/or floor effect. Despite the absence of explicit domain knowledge, the derived mapping captured spatial relationships that align with clinical expectations.

As illustrated in Figure 4B, D, the group t-statistic maps indicated that the structural locations with the most significant impact on VF sensitivity prediction were largely consistent with the Garway-Heath map. However, some minor discrepancies were observed. For instance, in both the healthy-to-early-glaucoma and moderate-to-advanced-glaucoma groups, points 28 and 37 were slightly closer to the temporal aspect in our derived map, while point 43 was slightly closer to the nasal aspect.

These discrepancies primarily occurred at the edge points of VF clusters defined in the Garway-Heath map, suggesting the existence of finer clusters that were not captured by the coarse mapping. Additionally, several factors might have contributed to the observed differences. First, despite temporal smoothing of VF tests to mitigate noise, the remaining sensitivity variability of VF measurements could still hinder the model's ability to accurately characterize the structure–function relationship, leading to potential inaccuracies in the spatial mapping. Second, the coarse individual image registration method to generate group maps did not account for morphologic variables such as disc to foveola angle. While we mitigated interindividual variation by using a relatively large sample size, these variations could still contribute to the observed discrepancies between our map and the Garway-Heath map. Finally, differences in the sample population, including demographic and clinical characteristics, could also contribute to slight variations in the structure–function mapping. To tell whether the discrepancies are artifacts or new information requires further investigation.

Previous studies have reported that the correlation between structure and function varies with the severity of the disease.^20^, ^21^, ^22^, ^23^ In line with these findings, our study also demonstrated a connection between saliency and the severity of VF damage. Figure 6 illustrates the pointwise Pearson's correlation between saliency and VF MD for all subjects in the test set. It was observed that the saliency exhibited a negative correlation with VF MD, indicating a stronger association between saliency and MD when defects are more severe. As a result, the group t-statistic map of the healthy-to-early-glaucoma group appears less representative compared with that of the moderate-to-advanced-glaucoma group. Furthermore, this correlation leads to symmetric spatial patterns for the subgroups with superior and inferior defects, as depicted in Figure 5. This correlation also explains the highlighted areas in the less-defected hemifields in Figure 5. For example, in the superior defect group, highlighted areas on the saliency map corresponding to the inferior portion of the VF are also present. Note that these points are also with a deficit as the average VF sensitivities are close to or <20 dB. Thus, some highlighted areas in the opposite hemifield are expected. Moreover, the differences between the 2 hemifields proves the potential toward an application of the proposed group saliency map, that is phenotyping the structure–function map.

While the application of occlusion analysis to visualize the effects of OCT on VF prediction is not groundbreaking,^8^^,^^16^ the majority of these studies primarily center around confirming the accuracy of the proposed DL model. For instance, Christopher et al^8^ employed a DL model to predict averaged function measurements of VF sectors as defined in the Garway-Heath map. They utilized occlusion analysis to generate a structure–function map for individual cases. Although their map demonstrated specific sectoral structure–function relationships, such as the model's emphasis on superior ONH structures to predict function in the inferior and inferior nasal VF sectors, and vice versa, it was not specifically tailored to assess the broader trend of spatial mapping between structure and function. Its primary objective was to establish the validity of the proposed DL model on an individual case basis. Similarly, Kihara et al^16^ managed to derive a more refined occlusion-based structure–function map with 2 separate DL sub-models, able to provide VF sensitivity estimation from 2D circumpapillary OCT and infrared SLO images, respectively. However, the resulting 2D occlusion-based heatmaps on infrared SLO images remained specific to individual cases. Moreover, neglecting the 2D en face information brought by infrared SLO images, they only plotted the distribution of the circumpapillary angles at which the highest peak of the heatmap was located. Thus, their mapping remained angular and could not represent the overarching structure-to-function trend.

In another study, Mariottoni et al^7^ developed a DL-based spatial structure–function mapping by simulating localized peripapillary RNFL defects and feeding the resulting thickness profile into a pretrained convolutional neural network model. The identified pattern exhibited agreement with previous maps such that the RNFL defects simulated on the temporal superior and temporal inferior regions led to arcuate VF defects in the inferior and superior hemifield, respectively. However, it is important to note that the derived map remained sectoral in nature and was confined to the peripapillary sampling circle, lacking a comprehensive representation of the entire structure–function relationship. Also, their method relied on RNFL segmentation.

Our study generated comprehensive maps that were consistent with previously published ones, providing detailed spatial relationships between structure and function potentially free from bias. However, there are several limitations to consider in this study. First, the accuracy of the saliency maps heavily relies on the chosen model architecture and the training process. Varied models and datasets can produce divergent saliency maps, encompassing the inherent biases of the specific model architecture and the training data utilized. Uncommon defect patterns, such as early onset patterns, may not be well captured by the network during training, leading to underappreciation of certain structure–function relationships. Additionally, VF test results in patients with glaucoma are susceptible to random noise and subjectivity,^24^, ^25^, ^26^ which inherently reduces the accuracy of the model in predicting VF sensitivities. Second, our technique relies on the assumption of a linear correlation between a region's significance and the fluctuation in the model's predictions when that region is occluded. However, DL models, especially those handling high-dimensional data such as 3D OCT images, may exhibit nonlinear relationships that simple occlusion fails to capture. Moreover, the spatial interconnections between different regions in a 3D OCT scan are not adequately addressed by the occlusion method. This oversimplification hinders a comprehensive understanding of the intricate interactions among various regions in the OCT scan. Furthermore, the choice of occlusion method (e.g., replacement with a gray patch, mean pixel value, or random noise) can impact the analysis outcomes, resulting in diverse interpretations of the model's behavior. Without a clear awareness of the limitations inherent in our proposed technique, there is a potential risk of overinterpreting the saliency maps generated. This overinterpretation could lead to misleading conclusions regarding the structure–function relationship. Another limitation is the use of naive registration in this study. Although the derived map demonstrates fine resolution and aligns well with known clinical knowledge and understanding of structure–function relationships, the naive registration does not account for refractive errors or other morphologic variables such as disc-foveola angle. These variations can contribute to differences in the spatial relationship between VF test points and corresponding ONH regions. Advanced registration techniques are required to uncover more subtle spatial relationships. Finally, we followed the conventional way of visualizing this relation as x-y projection to collapse information along the z-axis so that clinicians will better appreciate the results, ignoring the fact that the saliency data we derived were essentially 3D data. Though such collapse partially compensates for the individual variability, it prevents us from deriving more comprehensive 3D structure-to-function mapping. The z-axis information might provide new insights. However, at this moment, we do not have clear explanation for the z-axis information. Thus, further investigation is needed to fully leverage the 3D nature of the ONH and interpret the z-axis information.

This study is the first work to utilize 3D OCT image data without segmentation around the ONH aiming for establishing VF-pointwise structure–function mapping. The revealed en face spatial correlations offer detailed and specific mapping that is consistent with previous studies, highlighting the potential of machine learning in establishing intricate structure–function relationships. The proposed DL methods are fully automated, data-driven, and able to utilize depth information. Combining with advanced registration methods, it is possible to fully unlock the 3D potential of OCT images and derive more comprehensive mappings. This opens up the possibility of discovering new structure–function relationships and lots of potential use of the proposed group saliency map such as phenotyping the structure–function map.
