# Supplementary material for: Deep-Learning-Based Group Pointwise Spatial Mapping of Structure to Function in Glaucoma
Source: Ophthalmol Sci. 2024 Apr 2;4(5):100523. doi: 10.1016/j.xops.2024.100523 (PMC11179402; doi:10.1016/j.xops.2024.100523)
Supplement: Appendix [file mmc1.docx]

**Appendix**

Figure 7 and Figure 8 show an example of a saliency volume corresponding to the VF test point 21. The videos showing the whole saliency volume are available via <https://youtu.be/2PnjkFtmBL8> (cross-sectional view) and <https://youtu.be/ZKJw4l64IYw> (enface-view).

To test the influence of within-subject correlation, we also plotted group t-statistic maps by randomly sampled one visit from one eye. 207 eyes were included for the healthy-to-early-glaucoma group while 66 eyes were included for the moderate-to-advanced-glaucoma group. The maps are shown in Figure 9. The group t-statistic maps are very similar in both Figure 4 and Figure 9, but Figure 9 were generated with fewer data points.
